# Contribution of Dental Amalgam to Urinary Mercury Excretion in Children: Woods et al. Respond

**DOI:** 10.1289/ehp.11013R

**Published:** 2008-03

**Authors:** James S. Woods, Michael D. Martin, Timothy A. DeRouen

**Affiliations:** Department of Environmental and Occupational Health Sciences, University of Washington, Seattle, Washington, jwoods@u.washington.edu; Department of Oral Medicine, University of Washington, Seattle, Washington; Department of Dental Public Health Sciences, University of Washington, Seattle, Washington

We are pleased to respond to Iavicoli and Carelli’s comments on our article (Woods et al. 2007).

Iavicoli and Carelli question our use of creatinine adjustment; in previous studies, we addressed the pros and cons of this issue in depth (Heyer et al. 2007; Martin et al. 1996; Woods et al. 1998). In terms of the present study (Woods et al. 2007), instead of advocating one approach over the other, we presented data, where appropriate (e.g., Figure 2), as both adjusted and unadjusted measures to provide the reader access to both sets of results. Iavicoli and Carelli note only a slight difference in Figure 2 between comparisons of adjusted and unadjusted urinary Hg levels at year 7; in our article we explained the higher variability (not bias) in the unadjusted values. Because creatinine adjustment did not otherwise alter the urinary Hg findings in the study, as stated in our Figure 3 (Woods et al. 2007), we did not present adjusted values for data described in Figures 3 or 4. Iavicoli and Carelli’s comments about blood Hg levels are not relevant because we did not measure blood Hg concentrations in this study.

Regarding Iavicoli and Carelli’s comment on “steady state,” data presented in our Figure 4 (Woods et al. 2007) allow the reader to discern the influence of additional amalgam treatment on Hg body burden over time, as inferred from annual urinary Hg levels. A comprehensive pharmacokinetic evaluation of Hg body burden was neither intended nor within the scope of this study.

Iavicoli and Carelli speculate that Hg sulfide on teeth surfaces might have affected observed changes in urinary Hg levels; however, others (Brune 1986; Brune and Evje 1985; Gebel and Dunkelberg 1996) have clearly shown that sulfide (and other oxidation) layers are continuously removed by the effects of mastication, as well as by hot foods and liquids. These layers do regenerate but are in a constant state of flux. Because all of the amalgam used in this study (Woods et al. 2007) was of a single formulation, there would have been no variation in the tendency to form sulfide layers from amalgam treatment. Additionally, although there may have been some variation in oral pH among study subjects that could have influenced this process, Hg elimination still occurs at a relatively constant state. Therefore, we do not consider Hg sulfide film formation to be a significant factor in urinary Hg excretion over time.

Iavicoli and Carelli question our use of “dose effect” to describe the relationship of amount of amalgam treatment received with urinary Hg concentration. However, we consider this depiction appropriate.

Finally, Iavicoli and Carelli point out that the trend that children who received up to nine initial amalgam fillings but no subsequent treatment returned to pretreatment values within 1 year is not clear. This statement should have been “within 1 additional year” (i.e., by year 3 of follow-up). Urinary Hg levels were highest approximately 2 years after initial amalgam treatment for those with no subsequent treatment; those with ≥ 10 fillings at initial treatment and no subsequent treatment took > 3 years (approximately 5 years) to return to pretreatment levels. For Figure 4 (Woods et al. 2007), we obtained confidence intervals, but they were not included because of the clutter they created in the figures. Thus, the statements refer to trends and not statistical significance.
